# Efficient Video Watermarking Algorithm Based on Convolutional Neural Networks with Entropy-Based Information Mapper

**DOI:** 10.3390/e25020284

**Published:** 2023-02-02

**Authors:** Marta Bistroń, Zbigniew Piotrowski

**Affiliations:** Institute of Communication Systems, Faculty of Electronics, Military University of Technology, 00-908 Warsaw, Poland

**Keywords:** CNN, entropy, information mapping, neural networks, watermarking, video watermarking, YUV

## Abstract

This paper presents a method for the transparent, robust, and highly capacitive watermarking of video signals using an information mapper. The proposed architecture is based on the use of deep neural networks to embed the watermark in the luminance channel in the YUV color space. An information mapper was used to enable the transformation of a multi-bit binary signature of varying capacitance reflecting the entropy measure of the system into a watermark embedded in the signal frame. To confirm the effectiveness of the method, tests were carried out for video frames with a resolution of 256 × 256 pixels, with a watermark capacity of 4 to 16,384 bits. Transparency metrics (SSIM and PSNR) and a robustness metric—the bit error rate (BER)—were used to assess the performance of the algorithms.

## 1. Introduction

The issue of copyright protection is a multi-billion-dollar problem affecting both developed and developing countries. Related to this is the phenomenon of multimedia piracy, i.e., the unauthorized distribution and redistribution of multimedia content such as films, TV programs, or audio files [1]. One of the main drivers of this phenomenon is the desire of consumers to watch new content as soon as it is released, without having to pay for premium TV services. The most common method for the illegal use of films and TV series is through stream ripping, while many consumers also illegally download and stream content [2]. Multimedia piracy is a source of huge financial losses for both owners and distributors of entertainment content and for consumers, as it is the source of many restrictions and limitations on offering entertainment to end customers [3].

The solution to the problem is to embed an invisible, capacious, and robust watermark in the media content, allowing the owner of the content to be identified and the source of the data leak to be traced in the event of an unauthorized distribution occurring. Digital Rights Management aims to develop systems to counteract the use of digital data in a manner contrary to the will of the publisher. Digital watermarking is very often used in this area [4,5,6].

A watermark embedded in the video content should comply with three basic paradigms to implement the method in commercial applications:

- Transparency, i.e., the invisibility of the watermark to the human visual system (HVS) [7]. The video viewer usually does not have access to the original video (without the watermark), so seeing minor modifications is impossible, but despite this, the watermark may not significantly affect the quality of the video, which is verified in a measurable way based on metrics [8].

The bit capacity—the number of watermark bits that can be encoded in one video frame—should be as high as possible to encode as much data as possible. Information mappers are used to transform the binary signature into a watermark [9,10].

The robustness of the algorithm is determined by its ability to decode the correct watermark from the material. Measurably, robustness is assessed based on the BER (bit error rate) metric by comparing the original watermark and the watermark extracted from the video [11].

The area of watermark embedding and extraction in video files has so far been dominated by three main approaches—watermark embedding in the spatial domain [12,13,14]; the transform domain with the Discrete Cosine Transform (DCT) [15], Discrete Wavelet Transform [16], Discrete Fourier Transform [17], or combined methods [18,19]; and the compression domain [20,21]. Currently, classical methods are increasingly being supported or replaced by the use of deep neural network algorithms [22], which have found applications in many areas, both civil and military [23]. Algorithms using neural networks are characterized by much higher efficiency, but also high computational complexity, which significantly increases the processing time of a single frame for watermark embedding [24].

This paper presents a modification of an algorithm using artificial neural network architectures to embed a static image in a luminance channel in the YUV color space with high transparency preserved. Modifications were made to the loss function and the model training process, and the architecture was adapted to handle five-dimensional input data (video sequences). In addition, an information mapper and a demapper have been implemented to allow high-capacity binary signatures to be encoded in the video frame. The main research problem addressed in the study was the selection of the algorithm’s hyperparameters to achieve the highest possible binary watermark capacity while maintaining high transparency and robustness.

The main contributions of this work are as follows:

The implementation of an entropy-based information mapper and demapper enabling the transformation of a binary signature into a watermark (and vice versa) for embedding and extracting the watermark in a video signal.

The modification of the watermarking algorithm in terms of extending the loss function and changing the learning procedure to ensure the high transparency and robustness of the method.

The laboratory testing of the watermarking algorithm for watermark capacities from 4 to 16,384 bits with the individual selection of optimal algorithm hyperparameters to confirm the efficiency and effectiveness of the method.

The remainder of the article is organized as follows: Section 2 presents the currently employed approaches for watermarking static images and video signals, Section 3 describes the method used and the modifications introduced, Section 4 presents the results of the experiments performed, and Section 5 summarizes the results of this work and provides directions for further research.

## 2. Related Works

**Figure 1:**
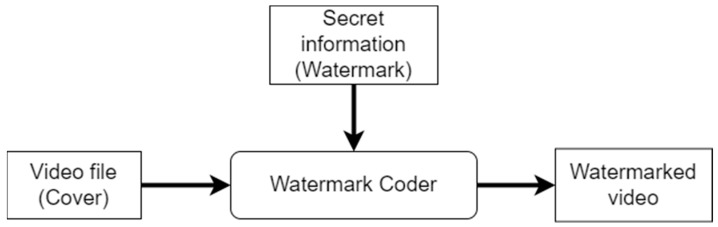
Watermarking diagram.

Digital watermarking is aimed at embedding a piece of specific information (watermark) in a media file, often called a cover, as shown in the diagram in Figure 1.

In classical methods, the watermark is transformed so that it is embedded in a selected domain, for example, the coefficients of a selected transform. The content with the embedded watermark is then restored to the original domain [25]. Currently, this approach is increasingly being supplanted by the use of deep neural networks, which adjust the weight of each layer of the network in the training process, enabling the creation of hierarchical representations of image features without the need to manually create such representations [26], to embed the watermark in an invisible and noise-resistant manner.

In [27], the authors proposed combining the wavelet tree method with a neural network. The luminance component in the YUV space is decomposed into wavelets to find a meaningful wavelet tree [28]. The correlation between the nodes of the wavelet tree is described by a non-linear relationship defined using a neural network. The solution made it possible to increase the transparency and robustness of the algorithm against typical attacks (rotation, Gauss filter, JPEG compression).

A similar approach to support the wavelet method through a neural network is presented in [29]. In the preprocessing procedure, the Arnold transform [30,31] and spline coding [32] were used, which were intended to make the watermark resistant and immune. The cover is transformed using DWT, and a trained neural network allows the embedding of the watermark in the wavelet domain to modify a small part of the input image and ensure high transparency.

One of the first approaches based solely on the use of deep neural networks is the algorithm described by Baluja [33]. The approach he proposed is based on an autoencoder architecture: one network (the encoder) is tasked with embedding the watermark, while the other (the decoder) extracts it from the image. In addition, the author used yet a third network (Prep Network), which prepares the watermark image for embedding. Preparation involves matching the watermark to the dimensions of the cover and transforming the image into a feature map using an edge and texture detector. All three architectures are trained during a single training procedure designed to optimize the defined loss function. The method allows the watermark to be embedded in all bits of the input image.

In [34], the authors added an adversary module to the autoencoder architecture. The algorithm is based on the idea of generative adversarial networks (GANs) described in 2014 [35]. The autoencoder acts as a generator, and it is mainly used to generate images with embedded watermarks and decode the image to obtain the watermark. The adversary is used to judge whether the image in the input is the original image or an image with an embedded watermark. They proposed a RivaGAN algorithm designed to embed a watermark in a video signal. To ensure a high degree of transparency, the authors enriched the generator and adversarial architecture with a custom attention mechanism that allows individual bits of the 32- or 64-bit watermark to be embedded in optimal areas of the cover. The attention mask produced by the encoder is also used by the decoder during the watermark extraction process. The authors also verified the robustness of the method against scaling, trimming, and MJPEG compression attacks.

Hao et al. [36] also proposed a solution based on the combination of an autoencoder and GAN architecture. Their main innovation was the addition of a high-pass filter before the discriminator to improve its sensitivity to high-frequency signal components. In addition, based on the assumption that the vision system pays more attention to the central area of the image, the penalty for the algorithm for modifying pixels in the central area was increased. The authors verified the effectiveness of the method for 64 × 64 pixel images by testing the robustness of the embedded watermark using basic attacks.

A significant disadvantage of marking algorithms based on neural networks is their high computational complexity. In [37], the authors proposed a number of optimizations to enable a learning procedure for high-resolution video signal marking algorithms. The method involves algorithmic and memory optimization for four neural architectures: a cover preparation network, a watermark preparation network, a watermark embedding network, and a watermark decoding network. An optimization of the batch normalization layer was applied, during which the number of calculations was reduced and the precision of the intermediate calculations was optimized to match the bit width of the processor dedicated to the calculations. The authors presented the effects of the hardware implementation in the proposed configuration.

Thanks to various techniques enabling the optimization of the operation of neural networks used to embed watermarks, a solution is presented in [38]. The authors used an approach based on an autoencoder architecture in an application designed to embed a watermark in screenshots taken with a mobile device. Instead of a preparatory network, the authors used a cosine transform and an inverse cosine transform to embed and extract the watermark in the DCT domain. The robustness of the method against basic attacks, such as blurring, Gaussian noise, rotation, scaling, edge sharpening, and JPEG compression, was shown through experiments.

## 3. Proposed Method

### 3.1. General Architecture of the Model

**Figure 2:**
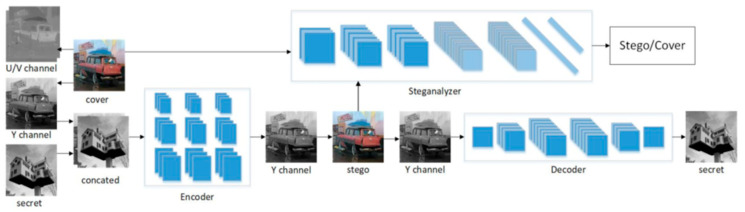
Block diagram of the ISGAN model—reprinted from [40].

The algorithm proposed in this paper is based on the use of an autoencoder built from convolution layers, combined with a discriminator to improve transparency and robustness metrics. The use of generative models described in [39] mainly determines the innovativeness of the described method and allows for the high performance of the algorithm. The approach is based on the ISGAN architecture for embedding a static image in another static image, described by Zhang, Dong, and Li [40]. The authors used three convolutional networks—a watermark encoder responsible for embedding a static grayscale image into the cover luminance channel, a watermark decoder to extract the embedded image, and an autoanalyzer acting as a discriminator to verify whether the image in the input is the original image or the embedded watermark image. A diagram of the algorithm is shown in Figure 2.

**Figure 3:**
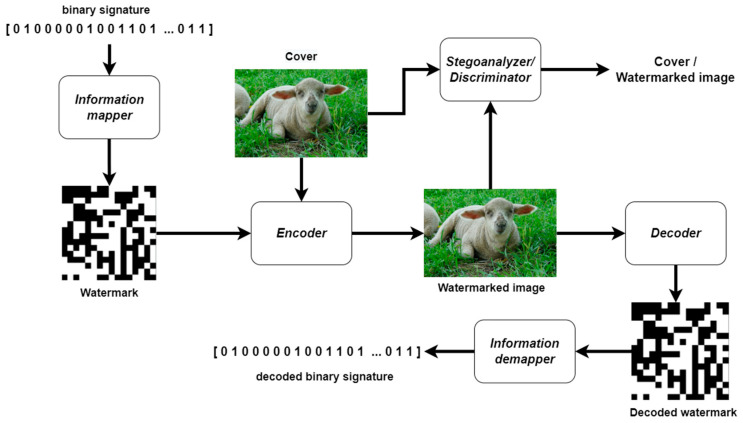
Block diagram of the proposed model.

**Figure 4:**
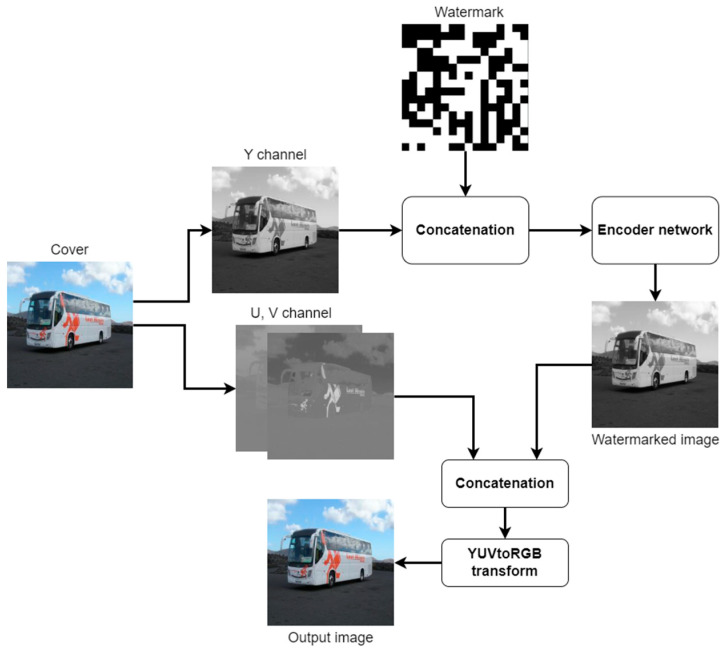
Block diagram of the encoder.

**Figure 5:**
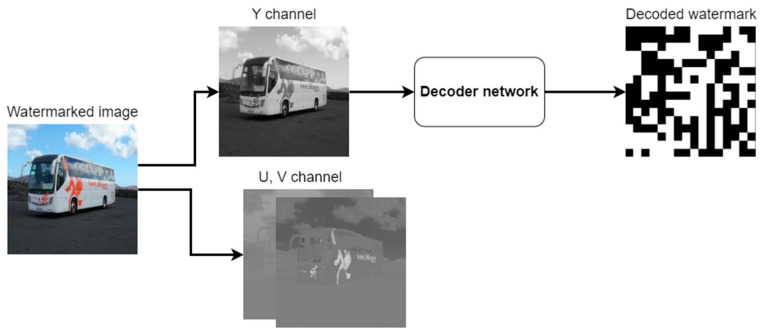
Block diagram of the decoder.

As part of the following work, the ISGAN architecture was extended with a mapper module upstream of the encoder and a demapper module downstream of the decoder, allowing a binary symbol with a certain number of bits to be converted into a static grayscale image and embedded in each video frame delivered to the encoder. The demapper performs the reverse operation: it converts the decoded grayscale image into a binary signature, which allows the robustness of the method to be unambiguously determined from the BER metric. In addition, the encoder, decoder, and discriminator architectures were adapted to the video-processing capabilities, i.e., processing 5-dimensional data tensors as video sequences. The block diagram of the proposed model with the modifications made is shown in the following figures: Figure 3, Figure 4 and Figure 5.

When the data enter the encoder, the size of the feature map is verified. A frame with dimensions of less than 1280 × 720 pixels is classified as standard definition (SD), while a frame with larger spatial dimensions is classified as high definition (HD). Depending on the dimensions, a transformation matrix is selected to convert the images from RGB space to YUV space. Converting an image from RGB to YUV allows data to be hidden only in the luminance channel, which does not carry any information about color. This makes it necessary to embed the watermark only in shades of gray, but it makes it easier to achieve the high transparency of the algorithm due to the modification of only one channel of the input image.

1. The processing of high-definition video frames is dedicated mainly to television broadcast applications; therefore, conversion to the appropriate color space should be performed in accordance with the International Communication Union (ITU) guidelines, which are described in the BT.709-6 standard. For this reason, there are two variants of the transformation matrix defined below. For standard definition:

(1) $$\begin{array}{c} Y\\ U\\ V \end{array}=\begin{array}{ccc} 0.299 & 0.587 & 0.114\\ -0.14713 & -0.28886 & 0.436\\ 0.615 & -0.51499 & -0.10001 \end{array}\times \begin{array}{c} R\\ G\\ B \end{array}$$

2. For high definition (according to the BT.709-6 standard):

(2) $$\begin{array}{c} Y\\ U\\ V \end{array}=\begin{array}{ccc} 0.2126 & 0.7152 & 0.0722\\ -0.09991 & -0.33609 & 0.436\\ 0.615 & -0.55861 & -0.05639 \end{array}\times \begin{array}{c} R\\ G\\ B \end{array}$$

The watermark prepared using the mapper is concatenated with the Y cover channel, and the resulting image is then processed by the convolutional network so that the encoding of the watermark is performed in an optimal way, i.e., taking into account transparency and robustness requirements. The image with the embedded watermark is again summed with the U and V chrominance components and then converted to RGB space using the appropriate transformation matrix to produce an output image in the standard color space used by end users.

1. For standard definition:

(3) $$\begin{array}{c} R\\ G\\ B \end{array}=\begin{array}{ccc} 1 & 0 & 1.13983\\ 1 & -0.39465 & -0.5806\\ 1 & 2.03211 & 0 \end{array}\times \begin{array}{c} Y\\ U\\ V \end{array}$$

2. For high definition (according to the BT.709-6 standard):

(4) $$\begin{array}{c} R\\ G\\ B \end{array}=\begin{array}{ccc} 1 & 0 & 1.28033\\ 1 & -0.21482 & -0.38059\\ 1 & 2.12798 & 0 \end{array}\times \begin{array}{c} Y\\ U\\ V \end{array}$$

In the decoder, the signal frame from RGB space is converted to YUV space, and the luminance channel is then processed by a convolutional network terminated by a sigmoid activation function. The resulting single-channel image is a decoded watermark.

The input and output data for the neural networks in the form of video sequences were defined as follows:

1. For the encoder:

Input:

cover = [N, C = 3, L, H, W], where N—batch size; C—number of channels; L—length of the video sequence (number of frames); H—height; W—width.

watermark = [N, C = 1, H, W]

Output:

watermarked image = [N, C = 3, L, H, W]

2. For the decoder:

Input:

watermarked image = [N, C = 3, L, H, W]

Output:

decoded watermark = [N, C = 1, H, W]

3. For the discriminator:

Input:

cover/watermarked image = [N, C = 3, L, H, W]

Output:

out = [N, 1]

**Figure 6:**
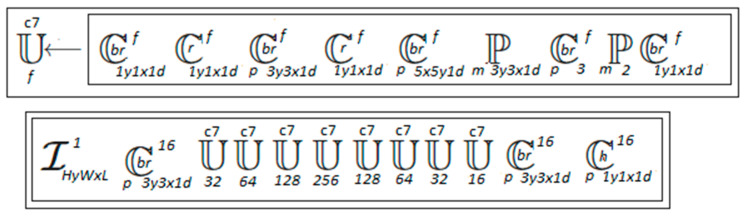
Encoder in symbolic notation.

**Figure 7:**
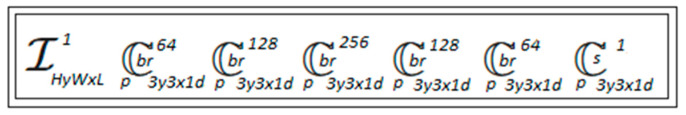
Decoder in symbolic notation.

**Figure 8:**
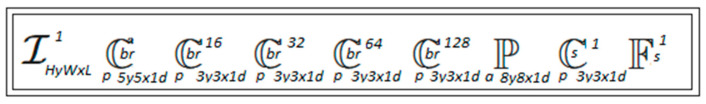
Discriminator in symbolic notation.

The fundamental architecture of the individual neural networks that make up the algorithm has not been changed. Adjustments were only made to adapt the model to work with the mapper and to process 5-dimensional sequences. Diagrams of the individual neural networks presented in symbolic notation according to [41] are shown in Figure 6, Figure 7 and Figure 8.

### 3.2. Mapper and Demapper of Information

**Figure 9:**
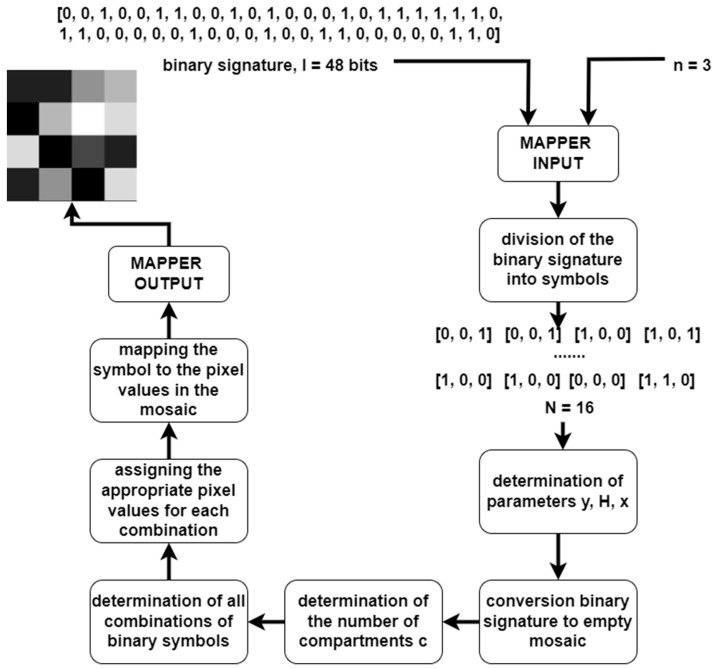
Diagram of the information mapper.

In practical applications of video watermarking, the embedded watermark is intended to carry a certain amount of information, e.g., about the owner of the media content. Information about the owner of the media content is stored in a database and assigned to a particular binary signature. To embed such information, mapper and demapper modules were implemented. The mapper maps a binary signature of a specific length, identical to the binary capacity of the watermark, to a static image in the form of a 256 × 256 pixel mosaic. In the implemented algorithm, the length of the binary signature must be a power of 4 with an exponent from 0 to 8 multiplied by a number *n* representing the number of bits encoded in one binary symbol. It allows 1 × *n* to 65,536 × *n* bits to be embedded in a single 256 × 256 pixel signal frame. The idea of how the mapper algorithm works is shown in the diagram in Figure 9.

The module takes two parameters as input—a binary signature of length l and a number *n* defining how many bits of the binary signature are to be dedicated to encoding one symbol. Based on the value of *n*, the algorithm divides the sequence of bits into symbols to be encoded. Depending on the number of symbols *N*, the mosaic area is divided into squares of *y* × *y* pixels, where *y* is the spatial dimension of a single square in the mosaic, *y* = *H*/*x*; *H* is the spatial dimension of the image watermark, *H* = 256; and *x* is the number of squares along one side of the mosaic, *x* = $\sqrt{N}$.

**Figure 10:**
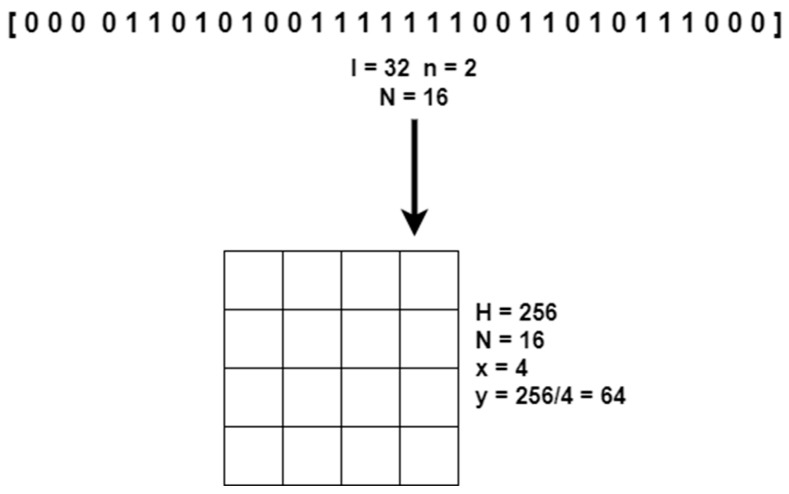
Conversion of a binary signature to a mosaic divided into squares.

An example of the conversion of a binary signature to a mosaic divided into squares is shown in Figure 10.

Depending on the number *n*, the number of compartments *c* to which each symbol will be assigned is determined, defined as follows:

(5) $$c=2^{n}$$

**Figure 11:**
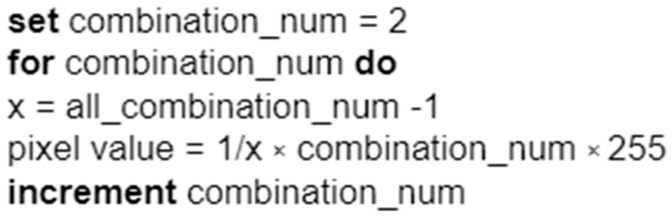
The idea behind the algorithm is that it assigns pixel values to individual bit symbols.

All possible combinations of 0 and 1 for a given value of *n* are determined using the Cartesian product and then sorted. For each combination, a pixel value is defined that will be assigned to the symbol in the given mapping. A value of 0 will always be assigned to the first symbol, and a value of 255 will always be assigned to the last symbol, corresponding to black and white pixel values. For more compartments, pixel values are defined according to the algorithm shown in the diagram in Figure 11.

**Figure 12:**
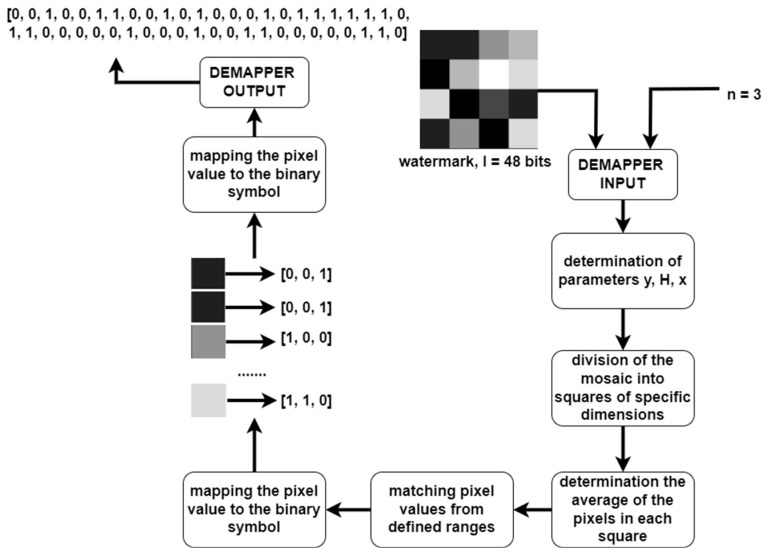
Diagram of the information demapper.

The demapper works in reverse: it converts the decoded mosaic watermark into a binary signature. The next steps performed by the demapper module are described in the diagram in Figure 12.

**Figure 13:**
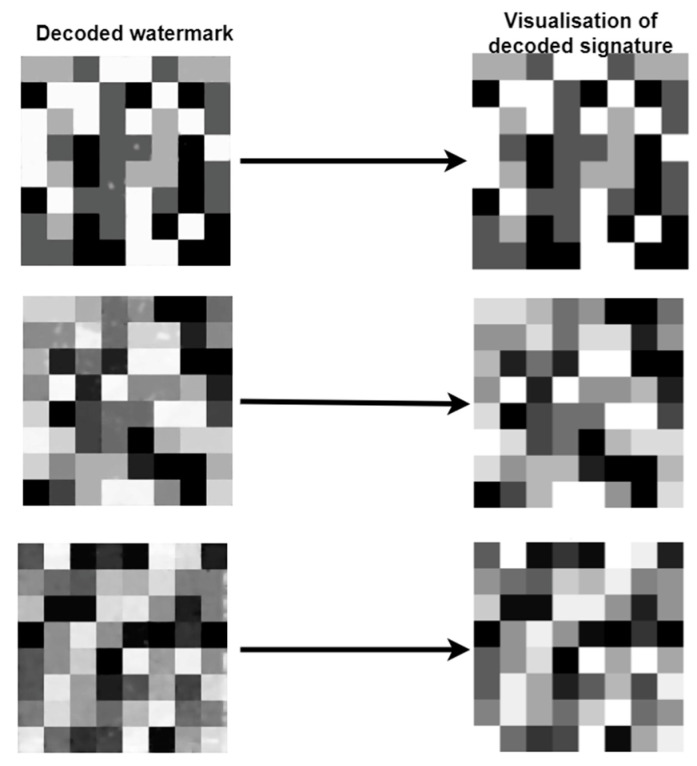
Three different examples of watermark signatures after decoding with various artifacts visible, and the results of averaging the pixel values during the decoding process to remove artifacts and error-free decoding.

The module takes two parameters as input—a watermark and a value defining the number of bits per symbol *n*. By analogy with the mapper, the *y*, *H*, and *x* values are determined. Based on the determined parameters, the mosaic is divided into squares, and then each square is separately decoded into a binary symbol. In the decoding process, the average pixel value for the area is determined, and then the nearest pixel value according to the predefined ranges is searched for based on this value. This ensures that it is not necessary for the decoder to decode the watermark without error, as it is possible to average out the interference that occurs in order for the mapper to decode the watermark correctly, as shown in the diagram in Figure 13.

A reverse mapping is then carried out. The mapper transforms the binary symbol into the pixel value in the given mosaic square, while the demapper transforms the decoded pixel value in the mosaic square into the binary symbol according to the adopted key. The sum of the symbols from all squares of the mosaic forms the final decoded binary signature.

The proposed information mapper and demapper are based on entropy. According to information theory, entropy (also called Shannon entropy) is a measure of the uncertainty associated with a random variable. The Shannon entropy equation estimates the average minimum number of bits needed to encode a sequence of symbols based on the frequency of the symbols:

(6) $$H\left(x\right)=-K\sum_{i=0}^{N-1}p_{i}log_{2}p_{i}$$

where *K* is a positive constant.

It follows from the equation that any operation to increase the number *N* and equalize the values of the probabilities *p* results in an increase in entropy [42]. In the implemented mapper, regardless of the number of bits per symbol *n*, the probability of the occurrence of each symbol is always equal for a given *n*. For example, if *n* = 1, *p* = 0.5 for symbol 0 and *p* = 0.5 for symbol 1, and if *n* = 2, *p* = 0.25 for each of the symbols: 00, 01, 10, and 11. This means that as the number of bits in the binary signature *N* and the number of bits per symbol *n* increase, the entropy of the watermark increases. As shown in [43], the background of images influences human visual perception. A single texture feature can be easily noticed by the viewer, but when the texture concerns a more complex image, it can be difficult to detect. The complexity and uncertainty of the original image alter the visual perception threshold of the target image, a phenomenon described in 1997 by Watson et al. [44] and termed entropy masking. Entropy is higher where the complexity and uncertainty of the image are greater. This leads to a reduction in the sensitivity of these areas, so the threshold for their perception increases accordingly, facilitating the transparent embedding of the watermark. On this basis, the following paper assumes that with the increase in the entropy of a watermark signature, its transparent embedding will be easier to obtain than in the case of watermarks with much lower entropy, which is verified in Chapter 4.

**Figure 14:**
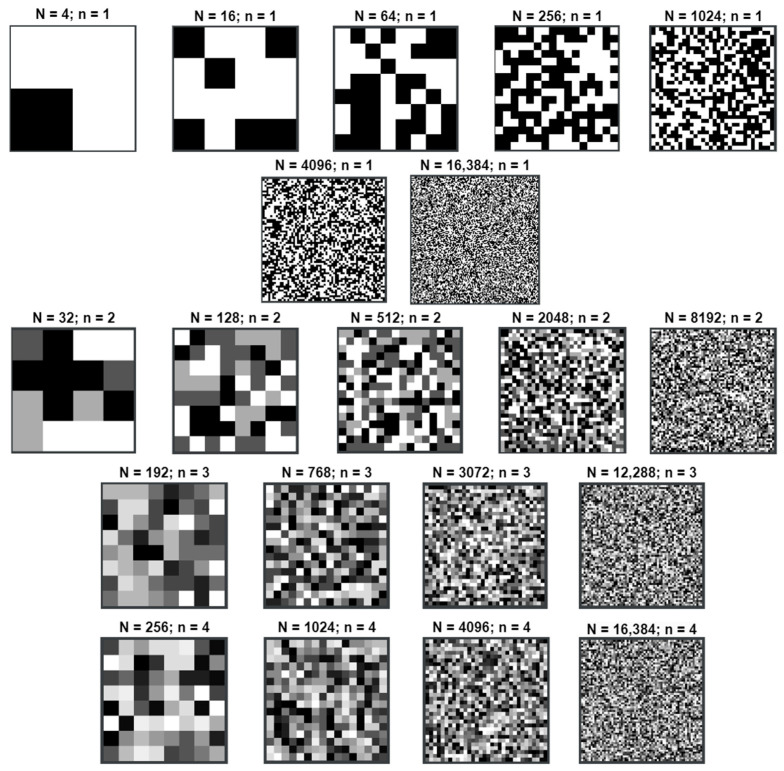
Examples of generated watermarks for all tested values of *N* and *n*.

Below are examples of watermarks with increasing entropy obtained using the implemented mapper for different values of the parameters *N* and *n* (Figure 14).

### 3.3. Algorithm Training Procedure

Based on a literature review of watermark embedding in static images and video signals [34,36], it was decided to use a two-step training procedure to achieve higher algorithm performance. One training epoch of the discriminator was performed first, followed by one training epoch of the generator (watermark encoder and decoder). The implementation of the learning process for both modules used the Adam optimizer [45] with a learning rate of *lr* = 0.0001, which is often used in the literature to optimize multivariate objective functions.

A standard approach used in generative adversarial networks was used to optimize the discriminator:

(7) $$L_{discriminator}=\min\left[-\left[\log\left(D\left(x\right)\right)+\log\left(1-D\left(G\left(x,s\right)\right)\right)\right]\right]$$

where 

*D* is the discriminator; 

*G* is the generator (watermark encoder and decoder); 

*x* is the cover; *s* is the watermark; 

and *G(x*, *s*) is the watermarked image.

An aggregate loss function taking into account the generator error, encoding error, and decoding error using appropriate weighting factors was used to optimize the watermark encoder and decoder module. The generator loss function was defined as follows:

(8) $$L_{genarator}=\min\left[\log\left(1-D\left(G\left(x,\;s\right)\right)\right)\right]$$

The optimization of the encoder is based on the use of a loss function described in [40], taking into account 3 measures of image similarity, i.e., the mean square error (MSE), similarity index (SSIM), and multi-scale structure similarity index (MS-SSIM), which allows the high transparency of the watermarked image:

(9) $$L_{enocoder}=\lambda_{a}\left(1-SSIM\left(x,\;x^{\prime }\right)\right)+\left(1-\lambda_{a}\right)\left(1-MSSSIM\left(x,\;x^{\prime }\right)\right)+\lambda_{c}MSE\left(x,\;x^{\prime }\right)$$

where *x* and *x*’ are the cover and watermarked cover; 

*λ_a_* and *λ_c_* are weighting factors for similarity metrics.

The above function was also used to optimize the decoder but supplemented with a lossy mapper, taking into account that the watermark is not required to decode the binary signature without error. The sum of both start functions gives the final decoder start function:

(10) $$L_{decocoder\_visual}=\lambda_{a}\left(1-SSIM\left(s,s^{\prime }\right)\right)+\left(1-\lambda_{a}\right)\left(1-MSSSIM\left(s,s^{\prime }\right)\right)+\lambda_{c}MSE\left(s,s^{\prime }\right)$$

(11) $$L_{decocoder\_mapper}=MSE\left(seq,\;seq^{,}\right)$$

(12) $$L_{decocoder}=L_{decocoder\_visual}+\lambda_{d}L_{decocoder\_mapper}$$

(13) $$L=L_{enocoder}+\lambda_{b}L_{decocoder}+\lambda_{e}L_{generator}$$

where *s* and *s*’ are the watermark and decoded watermark; 

*seq* and *seq^’^* are the binary signature and decoded binary signature; 

and *λ_b_, λ_d_,* and *λ_e_* are weighting factors taking into account the contribution of individual elements to the final loss function coding and decoding modules. 

## 4. Results and Discussion

### 4.1. Metrics

The efficiency of the algorithm was tested for the value of the number of bits embedded in the image *N* in the range from 4 to 16,384 bits at various values of the number of bits per one symbol *n*. For each variant, training and validation of the developed neural network algorithm were carried out together with the selection of the optimal values of hyperparameters and weighting factors. The purpose of selecting the parameters was to obtain an algorithm that would allow the embedding of a specific number of binary signature bits in the image in a transparent and robust manner at the same time. The fulfillment of the conditions was verified during the validation epochs using the PSNR and SSIM metrics for transparency and the BER metric for robustness. The metrics are defined below:

- Luminance comparison function: *x* and *y* represent the two images being compared, while *μ* represents the average value. *C*_1_ is the stability constant when the denominator is 0, calculated as *C*_1_ = 0.01^2^:

(14) $$l\left(x,y\right)=\frac{2\mu_{x}\mu_{y}+C_{1}}{\mu_{x}^{2}+\mu_{y}^{2}+C_{1}}$$

- Contrast comparison function: σ is the standard deviation for a given image, while *C_2_* is a constant value, equal in calculations to *C*_2_ = 0.03^2^:

(15) $$c\left(x,y\right)=\frac{2\sigma_{x}\sigma_{y}+C_{2}}{\sigma_{x}^{2}+\sigma_{y}^{2}+C_{2}}$$

- Structure comparison function: *C_3_* is a constant whose value in the calculations was assumed to be equal to *C*_3_ = *C*_2_/2:

(16) $$s\left(x,y\right)=\frac{\sigma_{xy}+C_{3}}{\sigma_{x}\sigma_{y}+C_{3}}$$

- SSIM—structural similarity index: coefficients *α*, *β*, and *γ* are weighting factors for each defined function; *α* = *β* = *γ* = 1 was assumed in the calculations:

(17) $$SSIM\left(x,y\right)={\left[l\left(x,y\right)\right]}^{\alpha }\cdot {\left[c\left(x,y\right)\right]}^{\beta }\cdot {\left[s\left(x,y\right)\right]}^{\gamma }$$

- MSE—mean square error: *m* and *n* are the row and column numbers in the image:

(18) $$MSE=\frac{1}{MN}\sum_{n=1}^{N}\sum_{m=1}^{M}{\left(x\left(n,m\right)-y\left(n,\;m\right)\right)}^{2}$$

- PSNR—Peak Signal-to-Noise Ratio: in the calculations, the value *R*^2^ = 2 was assumed:

(19) $$PSNR=10log_{10}\frac{R^{2}}{MSE}$$

- BER—bit error rate: the ratio of the number of bits decoded incorrectly *bit_err_* to all decoded bits *bit_all_*:

(20) $$BER=\frac{bit_{err}}{bit_{all}}$$

### 4.2. Results

The Pascal VOC Dataset [46] with over 17,000 training samples was used to train the algorithms. Each training of the algorithm consisted of 25 or 30 epochs. The algorithms were developed using Python and the PyTorch deep learning framework. During training, two Nvidia GeForce RTX 3090 graphics processors were used to speed up the learning process. The following table (Table 1) shows all training variants that were started and successfully completed.

In the case of variants with one bit per encoding of each symbol, the values of weighting factors were universal and correct for all values of *N*. For larger values of the parameter *n*, it was necessary to individually select the value of *λ_b_* for each case and to reduce the value of *λ_d_* to 0.6. The values were selected in an empirical way: training was carried out with the modification of the coefficients until optimal results were obtained. The changes resulted from the need to place more emphasis on optimizing the decoder start function in order to obtain the required algorithm robustness.

Changes in the parameter values were not intended to affect the results of individual algorithms. The modification of the loss function coefficients was necessary due to the impossibility of obtaining convergent training with incorrectly selected parameters. Each variant of the number of bits is a separate algorithm that requires the individual selection of parameters.

The number of epochs was initially set at 25, and during the research, it was decided to increase it to 30 due to the need to check whether there is any deterioration of transparency during training, which appeared with the increase in the epoch number with poorly selected training parameters (coefficients for the loss function).

**Figure 15:**
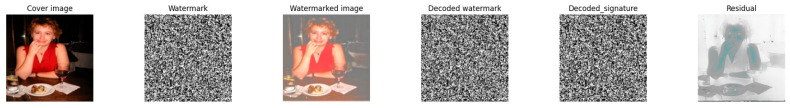
Failure to embed 32,768 bits. The following images show the cover, watermark, watermarked image, decoded watermark, visualization of decoded signature, and the picture of the difference between cover and watermarked images (Residual).

The maximum number of bits that could be encoded was 16,384 bits; in the case of higher values, it was impossible to obtain the transparency of the algorithm regardless of the choice of weighting factors (Figure 15).

**Figure 16:**
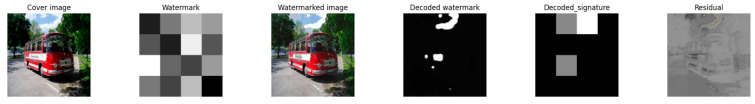
Failure to embed 64 bits when encoding one symbol using 4 bits.

When increasing the value of the parameter *n*, it was necessary to increase the minimum value of the number of bits *N* (increasing the entropy of the watermark), because it was impossible to obtain the required resistance of the watermark regardless of the choice of weighting factors (Figure 16).

The table below presents the values of the loss function and the BER metrics obtained during the training of individual variants (Table 2).

When binary signatures were encoded using 1 bit per symbol, it was possible to obtain BER values close to 0 (about 0.002), except for the first case, where only 4 bits were encoded. With the increase in the *n* parameter, it was more difficult to maintain the high robustness of the algorithm. The BER value during the training increased to about 0.005 for *n* = 2, from 0.015 to 0.044 for *n* = 3, and from 0.080 to even 0.113 for *n* = 4. The value of the encoder loss function, which determines the final transparency of the method, decreased with the increasing resolution of the mosaic of the watermark. The lowest results were achieved for resolutions of 4x4 for *n* = 1, 64 × 64 for *n* = 2 and for *n* = 3, and 32 × 32 for *n* = 4. In the case of the very low resolution of the mosaic, transparent watermark embedding was difficult or impossible, as described at the beginning of the chapter.

**Figure 17:**
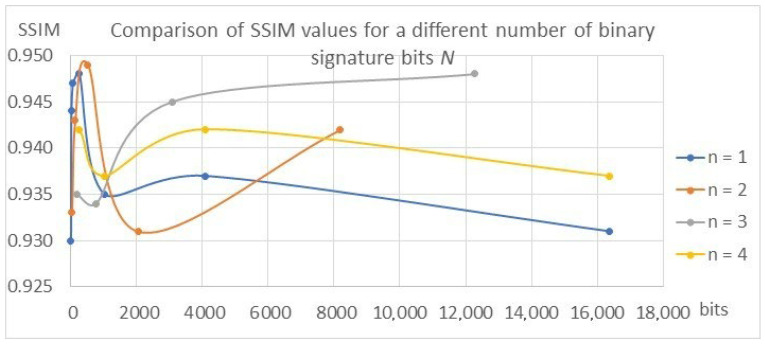
Comparison of SSIM metric values.

**Figure 18:**
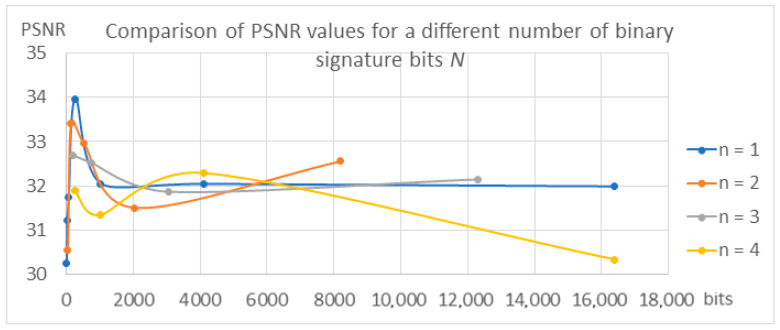
Comparison of PSNR metric values.

**Figure 19:**
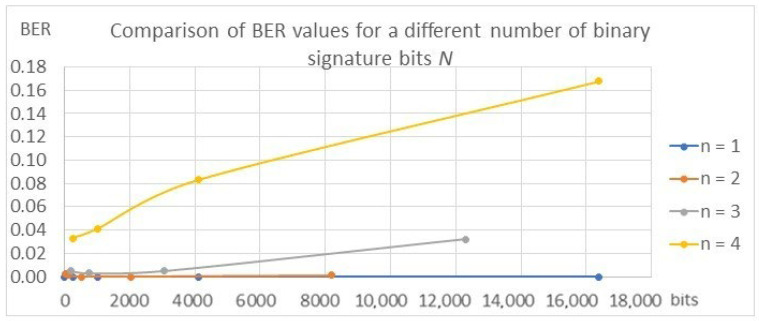
Comparison of BER metric values.

The results confirming the described dependencies were also obtained during the validation of the models for individual variants, which are presented in Table 3 and in Figure 17, Figure 18 and Figure 19.

The values of the SSIM and PSNR transparency metrics are similar for all tested variants and range from 0.93 to 0.95 for SSIM and from 30 to 34 for PSNR, which proves the achievement of the high transparency of the algorithms, which ensures the condition of the watermark being invisible to the recipient. In the case of BER metrics, the values for *n* = 1 and *n* = 2 oscillate around 0, which proves that the watermark was correctly decoded. With the increase in the parameters *n* and *N*, the BER value increases, reaching a value of 0.168 for the variant *N* = 16,384 bits and *n* = 4. This means that almost 17% of the bits, i.e., about 2785 bits in the signature, are incorrectly decoded, which means the disqualification of the marking algorithm or the need to use redundant coding.

### 4.3. Comparison with Other Algorithms

Most of the watermarking algorithms based on neural networks described in the literature deal with the problem of embedding a static image in another static image or in a video signal frame, which makes it impossible to compare the effectiveness of the algorithm at a given binary capacity. Below is a comparison of the method described in this article with the RivaGAN algorithm [34], which also distinguishes various variants of the embedded binary sequence (Table 4).

The accuracy of the model was determined as the inverse of BER, i.e., the ratio of the number of bits decoded correctly to all embedded bits. For both variants studied by the authors, i.e., 32 bits and 64 bits, our algorithm is characterized by worse transparency but higher accuracy. In the case of 64 bits, the SSIM metric values are similar (0.950 for RivaGAN and 0.947 for our algorithm). The table also shows a variant for which transparency was achieved at a level almost equal to the RivaGAN algorithm (SSIM = 0.949) with a precision of 1.0 and a much higher watermark capacity—512 bits. Our main goal was to find a balance between the transparency and resistance of the character with the largest capacity of the embedded binary sequence.

### 4.4. Discussion

The research results show that with the increase in watermark entropy, it is easier to obtain the high transparency of the method; however, with too high a complexity of the binary signature (over 16,384 bits), transparency is impossible to maintain. Meeting the robustness criterion is not possible with very low entropy of the watermark, especially when increasing the value of the parameter *n*; however, in the case of very complex watermarks encoded using many shades of gray, it is also not possible to decode the watermark without errors, which is caused by errors during the rounding of decoded bit values to the nearest interval defining the specified binary symbol. The use of the parameter *n* = 1 allows these errors to be limited to 0. However, the coding of very complex binary signatures using 1 bit is very computationally expensive. With the increase in the resolution of the watermark, the time required for the training and evaluation of individual algorithms is significantly longer due to the longer time required for mapping and demapping binary signatures. Table 5 compares the training times of algorithms with 1-bit symbols for different lengths of binary signatures.

To train the algorithm embedding 4096 bits, it was necessary to conduct the learning process for almost two days, while the training of the algorithm embedding the same number of bits when coding 4 bits per symbol lasted less than 20 h. It is necessary to find a compromise between the parameter *n*, which is the number of bits used to encode one symbol, and the efficiency of the algorithm, which will enable the development of a method characterized by efficiency and relatively low computational complexity. Encoding a larger number of bits is important for practical reasons because it allows an increase in the information capacity, which allows the encoding of a large amount of data regarding, for example, the owner of the content or the creation of a larger number of unique binary signatures, enabling the recording of a large number of various types of content.

### 4.5. Visualization of the Operation of the Model

The figures below show visualizations of the operation of each trained algorithm, showing the efficiency of watermark embedding and extraction for various video frames from the validation dataset (Figure 20, Figure 21, Figure 22, Figure 23, Figure 24, Figure 25, Figure 26, Figure 27, Figure 28, Figure 29, Figure 30, Figure 31, Figure 32, Figure 33, Figure 34, Figure 35, Figure 36, Figure 37, Figure 38 and Figure 39).

## 5. Conclusions

The problem of copyright protection in multimedia content, both audio and video, is currently a very popular issue analyzed by both researchers and commercial institutions developing ready-made DRM systems. Among the solutions used, watermarking is the dominant strategy, especially with the use of neural network algorithms, enabling the improvement of key watermarking paradigms, i.e., transparency, resistance, and bit capacity, to values impossible to achieve when using only classical methods of watermarking.

This article presents an algorithm for marking video signals based on the architecture of convolutional networks and the architecture of the GAN network, characterized by high transparency (SSIM above 0.93 and PSNR above 30) and robustness (BER metric value at the level of several percent for almost all analyzed variants). The main advantage of the presented algorithm is the use of an information mapper based on entropy that allows the embedding of complex, multi-bit binary signatures of up to 16,384 bits. Increasing the entropy of the watermark made it possible to obtain the high transparency of the algorithm, with a very high capacity at the same time. Each variant of the watermark signature (each pair of parameters N and n) was treated as a separate algorithm, for which the appropriate values of the weighting coefficients of the complex loss function were empirically selected, which allowed optimal results to be obtained.

The capacity of the tagging algorithm is important in the context of the commercial application of the method. The protection of copyright or content distribution rights requires marking the content with a complex watermark containing information about both the content and the owner. To encode such complex information, it is necessary to send a large number of bits.

The developed algorithm is the basis for further work in the field of watermarking. The next stage of work will be devoted to making the algorithm resistant to lossy compression using the latest video codecs, i.e., H.264 and H.265.

## Figures and Tables

**Figure 20:**
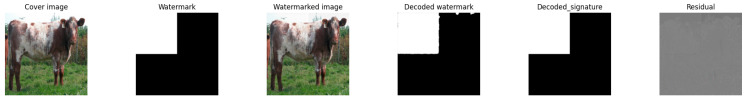
Visualization of the algorithm for the binary signature variant *N* = 4, *n* = 1.

**Figure 21:**
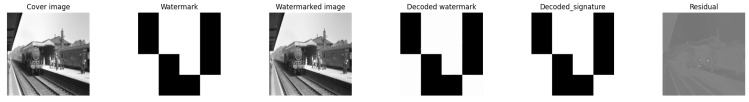
Visualization of the algorithm for the binary signature variant *N* = 16, *n* = 1.

**Figure 22:**
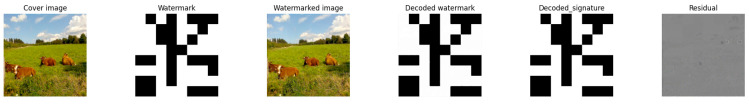
Visualization of the algorithm for the binary signature variant *N* = 64, *n* = 1.

**Figure 23:**
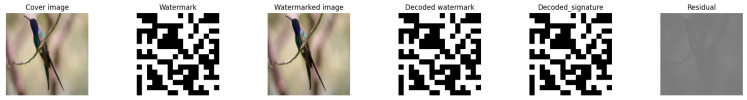
Visualization of the algorithm for the binary signature variant *N* = 256, *n* = 1.

**Figure 24:**
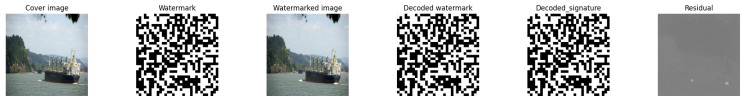
Visualization of the algorithm for the binary signature variant *N* = 1024, *n* = 1.

**Figure 25:**
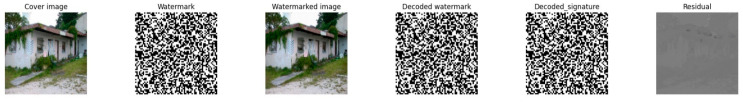
Visualization of the algorithm for the binary signature variant *N* = 4096, *n* = 1.

**Figure 26:**
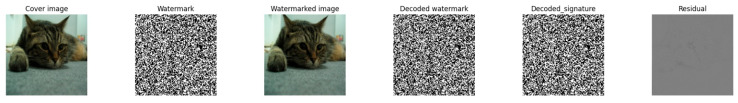
Visualization of the algorithm for the binary signature variant *N* = 16384, *n* = 1.

**Figure 27:**
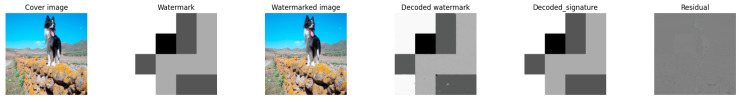
Visualization of the algorithm for the binary signature variant *N* = 32, *n* = 2.

**Figure 28:**
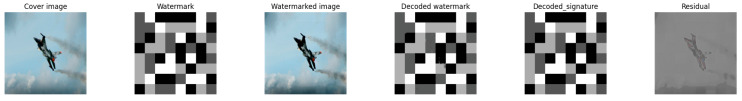
Visualization of the algorithm for the binary signature variant *N* = 128, *n* = 2.

**Figure 29:**
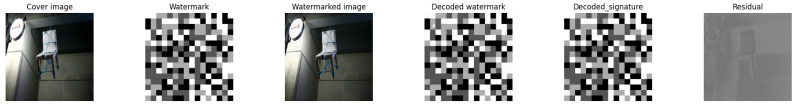
Visualization of the algorithm for the binary signature variant *N* = 512, *n* = 2.

**Figure 30:**
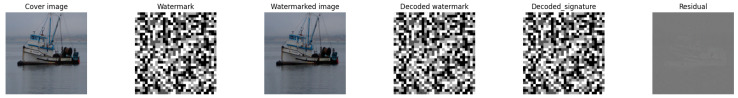
Visualization of the algorithm for the binary signature variant *N* = 2048, *n* = 2.

**Figure 31:**
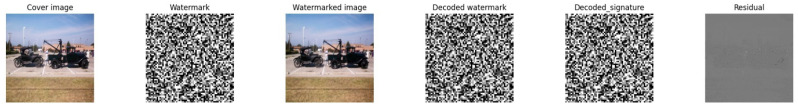
Visualization of the algorithm for the binary signature variant *N* = 8192, *n* = 2.

**Figure 32:**
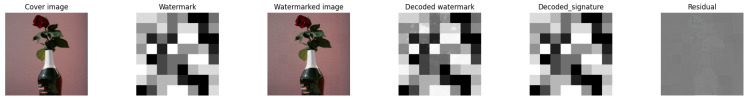
Visualization of the algorithm for the binary signature variant *N* = 192, *n* = 3.

**Figure 33:**
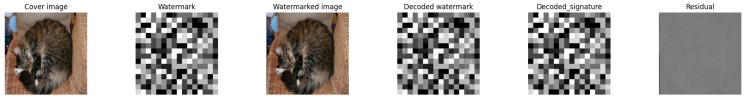
Visualization of the algorithm for the binary signature variant *N* = 768, *n* = 3.

**Figure 34:**
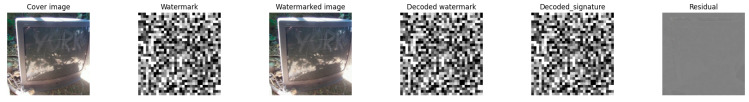
Visualization of the algorithm for the binary signature variant *N* = 3072, *n* = 3.

**Figure 35:**
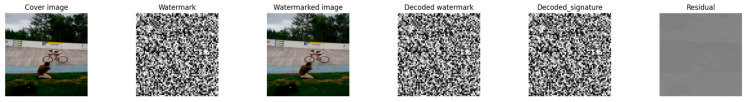
Visualization of the algorithm for the binary signature variant *N* = 12,288, *n* = 3.

**Figure 36:**
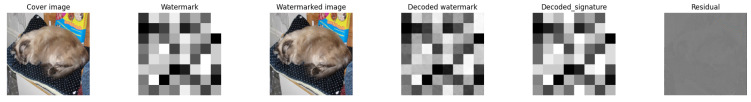
Visualization of the algorithm for the binary signature variant *N* = 256, *n* = 4.

**Figure 37:**
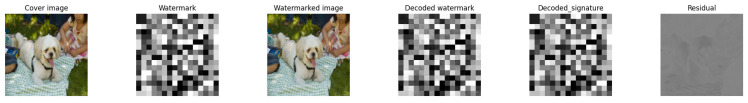
Visualization of the algorithm for the binary signature variant *N* = 1024, *n* = 4.

**Figure 38:**
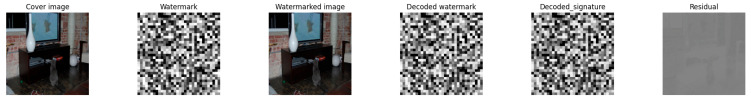
Visualization of the algorithm for the binary signature variant *N* = 4096, *n* = 4.

**Figure 39:**
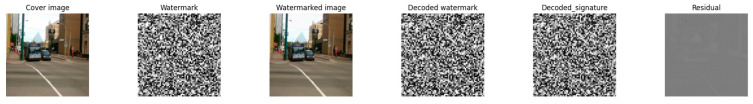
Visualization of the algorithm for the binary signature variant *N* = 16,384, *n* = 4.

**Table 1:** Variants used during model training.

Number of Binary Signature Bits *N*	Number of Bits to Encode One Symbol *n*	Value of Weighting Factor *λ_b_*	Value of Weighting Factor *λ_d_*	Number of Epochs
4	1	0.8	0.7	25
16	1	0.8	0.7	30
64	1	0.8	0.7	25
256	1	0.8	0.7	25
1024	1	0.8	0.7	25
4096	1	0.8	0.7	25
16,384	1	0.8	0.7	11 *
32	2	0.95	0.6	25
128	2	0.9	0.6	30
512	2	0.75	0.6	30
2048	2	0.75	0.6	30
8192	2	0.65	0.6	30
192	3	0.96	0.6	14 *
768	3	0.93	0.6	30
3072	3	0.85	0.6	30
12,288	3	0.8	0.6	30
256	4	0.97	0.6	30
1024	4	0.93	0.6	30
4096	4	0.8	0.6	30
16,384	4	0.8	0.6	30

* Early termination of the training due to a failure or a very long calculation time while obtaining results that meet the assumed criteria.

**Table 2:** Values of the loss function while training the algorithm.

Training Variant N_n	Discriminator Loss	Encoder Loss	Mapper Loss	Decoder Visual Loss	Decoder Loss	Generator Loss	Loss	BER
4_1	0.023	0.095	0.007	0.027	0.032	0.011	0.130	0.008
16_1	0.015	0.065	0.001	0.000	0.001	0.011	0.076	0.002
64_1	0.028	0.071	0.001	−0.002	−0.001	0.012	0.080	0.001
256_1	0.021	0.073	0.001	0.003	0.004	0.011	0.087	0.000
1024_1	0.011	0.078	0.001	0.007	0.008	0.013	0.096	0.000
4096_1	0.013	0.075	0.003	0.013	0.015	0.010	0.096	0.002
16384_1	0,030	0.091	0.003	0.009	0.012	0.010	0.110	0.002
32_2	0.019	0.074	0.004	−0.001	0.002	0.010	0.086	0.004
128_2	0.010	0.077	0.001	−0.004	−0.004	0.027	0.098	0.001
512_2	0,018	0.070	0.001	0.002	0.003	0.013	0.083	0.001
2048_2	0.017	0.079	0.005	0.018	0.021	0.011	0.105	0.005
8192_2	0.021	0.068	0.006	0.014	0.018	0.011	0.090	0.006
192_3	0.024	0.091	0.026	0.005	0.020	0.013	0.123	0.026
768_3	0.022	0.093	0.015	0.014	0.024	0.020	0.133	0.015
3072_3	0.015	0.082	0.044	0.043	0.070	0.010	0.151	0.044
12288_3	0.014	0.067	0.024	0.013	0.027	0.010	0.098	0.024
256_4	0.012	0.102	0.085	0.013	0.064	0.026	0.187	0.085
1024_4	0.019	0.075	0.080	0.018	0.066	0.010	0.145	0.080
4096_4	0.023	0.059	0.076	0.017	0.062	0.010	0.118	0.076
16384_4	0.015	0.072	0.113	0.018	0.086	0.011	0.150	0.113

**Table 3:** Summary of the SSIM, PSNR, and BER metrics and the value of the decoder loss function during the validation of individual variants.

Training Variant N_n	SSIM	PSNR	BER
4_1	0.930	30.256	0.000
16_1	0.944	31.223	0.001
64_1	0.947	31.738	0.002
256_1	0.948	33.962	0.000
1024_1	0.935	32.047	0.000
4096_1	0.937	32.040	0.000
16384_1	0.931	31.983	0.000
32_2	0.933	30.550	0.002
128_2	0.943	33.409	0.001
512_2	0.949	32.958	0.000
2048_2	0.931	31.498	0.000
8192_2	0.942	32.554	0.001
192_3	0.935	32.694	0.005
768_3	0.934	32.512	0.003
3072_3	0.945	31.856	0.005
12288_3	0.948	32.136	0.032
256_4	0.942	31.882	0.033
1024_4	0.937	31.338	0.041
4096_4	0.942	32.294	0.083
16384_4	0.937	30.328	0.168

**Table 4:** Comparison of the described method with another algorithm.

	RivaGAN 32 Bits	Our Model 32 Bits	RivaGAN 64 Bits	Our Model 64 Bits	Our Model 512 Bits
SSIM	0.960	0.933	0.950	0.947	0.949
accuracy	0.992	0.998	0.983	0.998	1.000

**Table 5:** Comparison of training time of algorithms.

Number of Bits	Training Time
4	9 h 5 min 50 s
16	9 h 14 min 34 s
64	9 h 39 min 14 s
256	11 h 20 min 7 s
1024	22 h 23 min 7 s
4096	44 h 12 min 34 s
16,384	54 h 38 min 41 s for 10 epochs

## Data Availability

Data available in a publicly accessible repository The PASCAL Visual Object Classes (VOC).
